# Impact of sociodemographic status and UTI symptoms on women’s health-care seeking and management in England: findings from an e-survey conducted during the first year of the COVID-19 pandemic

**DOI:** 10.3399/BJGPO.2023.0039

**Published:** 2023-10-18

**Authors:** Emily Cooper, Brieze Read, Leigh Sanyaolu, Haroon Ahmed, Donna Lecky

**Affiliations:** 1 Primary Care and Interventions Unit, UK Health Security Agency, Twyver House, Gloucester, UK; 2 Division of Population Medicine, Cardiff University, Cardiff, UK; 3 School of Medicine, Cardiff University, Cardiff, UK

**Keywords:** female, dysuria, prescribing, surveys and questionnaires, urinary tract infections, urology, primary healthcare, general practitioners

## Abstract

**Background:**

Multiple factors may influence women’s experiences of urinary tract infection (UTI) and its clinical management.

**Aim:**

To explore how women’s background, symptoms, and severity of symptoms influence UTI reporting and management.

**Design & setting:**

Internet questionnaire targeting women in England, focusing on UTI symptoms, care seeking, and management.

**Method:**

A total of 1096 women aged ≥16 years with UTI symptoms in the previous year completed the questionnaire in March and April 2021. Multivariable logistic regression was ued to estimate the odds of relevant outcomes while adjusting for background characteristics.

**Results:**

Women with children in their household, who were aged under 45 years, or who were married or cohabitating were more likely to experience UTI symptoms. The odds of antibiotic prescribing were lower if women reported dysuria (adjusted odds ratio [AOR] = 0.65, 95% confidence interval [CI] = 0.49 to 0.85), frequency (AOR = 0.63, 95% CI = 0.48 to 0.83), or vaginal discharge (AOR = 0.69, 95% CI = 0.50 to 0.96), but higher if reporting haematuria (AOR = 2.81, 95% CI = 1.79 to 4.41), confusion (AOR = 2.14, 95% CI = 1.16 to 3.94), abdominal pain (AOR = 1.35, 95% CI = 1.04 to 1.74), or systemic symptoms (AOR = 2.04, 95% CI = 1.56 to 2.69). Those with abdominal pain or two or more of nocturia, dysuria, or cloudy urine had lower odds of receiving a delayed antibiotic, while those with incontinence, confusion, unsteadiness, or low temperature had higher odds of a delayed prescription. Increasing symptom severity was associated with greater odds of receiving antibiotics.

**Conclusion:**

Except for reduced prescribing if a woman had dysuria and frequency, antibiotic prescribing followed an expected pattern, aligning generally with national guidance. Symptom severity and the likelihood of systemic infection probably influenced care seeking and prescribing. Sexual intercourse and childbirth may be key times to target women with messages about UTI prevention.

## Introduction

In 2019, the UK government set a national target to reduce gram-negative blood stream infections by 50% and reduce antibiotic resistant infections by 10%.^1^ To support this, national agencies developed tools and guidance for primary care to facilitate prevention, diagnosis, and management of urinary tract infections (UTIs).^2,3^ Research that underpins these resources often focuses on ways to improve UTI management.^3^ However, data show that women’s circumstances may influence UTI reporting, care seeking, and subsequent management.^4^

A 2014 cross-sectional household survey conducted with women in England found that 37% of women reported having a UTI in their lifetime.^4^ Most women (95%) reported contacting a health professional about their most recent UTI, with severe or persistent symptoms being the main reason for seeking care. Of women reporting any UTI, most contacted general practice (65%), the out-of-hours service (14%), a pharmacist (13%), or an online doctor (1%), and 74% reported being prescribed an antibiotic for their most recent UTI. Reporting prevalence varied by age group (36% for 16–34 years; 42% for 35–54-years; 33% for ≥55 years) and social class. The study was unable to look at relationships in detail while controlling for potential confounding variables.

Previously, tools for management of UTI focused on women seeking health care in person. Changes in care provision after the COVID-19 pandemic have influenced care seeking and provision of care. Qualitative data collected from primary care providers in eight European countries from April to July 2020 showed an increase in use of digital technology and remote consultations, with interviewees in some countries, including England, desiring a continuation of remote consultations for some groups of patients after the pandemic.^5^ Findings from a report published using data from the Royal College of General Practitioners Research and Surveillance Centre showed that by mid-March 2021 in England, telephone and video appointments still accounted for 54% of total appointments, while face-to-face appointments made up 46% of consultations.^6^

It is important to understand and acknowledge these factors in clinical guidance if UTI care seeking and outcomes are to be influenced. The aim of this study was to understand how women’s backgrounds and symptoms influenced their reporting and experience of UTIs during COVID-19, to ensure evidence-based national guidance and resources are developed that can support current clinical advice and decisionmaking. This will feed into a larger review of UTI diagnostic decision tools, which is currently underway, as well as overarching strategies to improve antibiotic management and reduce antibiotic resistance.^3^

## Method

The study was run by a market research agency (Ipsos) from 13 March to 13 April 2021, and employed an e-survey, multiple-choice questionnaire based on an earlier survey conducted in 2014 (Supplementary Appendix 1). The study targeted women aged ≥16 years in England through Ipsos’s online panels, which are continuously refreshed using multisource recruitment methods.

A questionnaire that had been used to conduct a similar survey in 2014 was adapted by the research team to include additional information specific to women’s symptoms and care seeking during the COVID-19 pandemic (Supplementary Appendix 1).^4^ This research team included healthcare staff (GPs, nurses, microbiologists), statisticians, and a behavioural science researcher. The symptoms reflected in the adapted questions were taken from national guidance, which included input from members of the public and other key stakeholders.^3^ UTI was defined at the outset of the questionnaire. Data on women’s backgrounds and household characteristics were collected. Women reporting UTI symptoms in the previous 12 months were asked for retrospective information about their symptoms and experiences.

Quotas were used for age, sex, region, social grade, and working status so that data could be weighted by these variables and to ensure representativeness. Sociodemographic variables were used to assess impact on self-reporting of a UTI in the previous year, and were also used to calculate adjusted odds ratios (AORs) (see Supplementary Appendix 2 for more information on how these variables were classified).

To explore whether symptoms aligned with current prescribing guidance in England and identify women with more severe infection, two additional symptom groupings were developed and used in the analysis: ‘strongly predictive symptoms’ (any two of dysuria, nocturia, or cloudy urine) and ‘system upset’ (any one of fever, flank pain, rigours/shaking, or a low temperature) (Supplementary Appendix 2). Strongly predictive symptoms were based on findings from research conducted on a cohort of women in England on identifying symptoms that were predictive of a positive urine culture.^7^ These symptoms align with the criteria for empirical treatment for antibiotics within national diagnostic guidance.^3^ Systemic upset was used to identify women who may have had a more severe infection or pyelonephritis.^3^ Vaginal discharge was included to investigate if women with this symptom were managed differently.^8^

To explore how women’s background characteristics influenced UTI reporting, two prevalence-based logistic models were run, the first focusing on any self-reported UTI in the previous 12 months (or not), and the second focusing on those who had recurrent UTI (>3) versus non-recurrent UTI (1–2) in the previous 12 months. Conditional odds were calculated by extending this model simultaneously to control for sociodemographic variables.

To analyse how symptoms influenced outcomes, logistic regression models were used to estimate crude odds ratios (ORs), AORs, and 95% confidence intervals (CIs). Data on symptom severity were collected using a 10-point Likert scale, and ordinal logistic regression was used to calculate ORs.

Sample weights based on women’s backgrounds and household characteristics were used in the analysis (Supplementary Appendix 2). The descriptive statistics and majority of the logistic regression modelling was conducted by two statisticians from the Ipsos team using SAS (version 9.4) and SPSS (version 28.0). The research team conducted some additional analysis specific to symptom severity using Stata (version 17), and were responsible for interpreting all of the analysis.

## Results

About 53 000 women aged ≥16 years in England were targeted through Ipsos’s online panels, of which 4153 completed the survey questionnaire (Table 1). About one-quarter of responders (26%, *n* = 1096) reported they had UTI symptoms in the previous year. Four per cent reported ≥3 episodes (*n* = 164). Women in the 25–34 age group represented the highest proportion of women reporting a UTI in the previous year (23%, *n* = 252) when compared with other age groups.

**Table 1.** Socioeconomic and demographic variables and women reporting a UTI in the previous year (weighted)

	Proportion of women reporting 'yes' to UTI in the previous year(*n* = 1096)	Proportion of women reporting ≥3 UTIs in the previous year(*n* = 164)	Proportion of responses for all responders(*n* = 4153)
≥1 UTIs in the previous year, *n (*%)	-	-	1096 (26.4)
Recurrent UTI (>3) in previous year, *n (*%)	-	-	164 (4.0)
**Demographic and background variables, *n* (%)**			
**Age, years**			
16–24	133 (12.1)	23 (14.3)	531 (12.8)
25–34	252 (23.0)	40 (24.1)	680 (16.4)
35–44	186 (16.9)	22 (13.3)	643 (15.5)
45–54	203 (18.5)	31 (18.9)	683 (16.5)
55–64	129 (11.8)	14 (8.8)	617 (14.9)
65–74	126 (11.5)	18 (11.2)	570 (13.7)
≥75	67 (6.2)	15 (9.4)	430 (10.3)
**Ethnicity, *n* (%)**			
White, UK	913 (83.4)	132 (80.9)	3367 (81.1)
White, other	75 (6.9)	11 (6.5)	308 (7.4)
Mixed ethnic group	23 (2.1)	2 (1.3)	69 (1.7)
Asian	47 (4.3)	10 (6.0)	191 (4.6)
Black	21 (1.9)	5 (2.9)	98 (2.4)
Other	9 (0.9)	3 (2.0)	25 (0.6)
No answer	7 (0.6)	1 (0.6)	96 (2.3)
**Social grade, *n* (%)**			
A/B	275 (25.1)	42 (25.4)	1048 (25.2)
C1	353 (32.3)	51 (31.3)	1312 (31.6)
C2	189 (17.3)	30 (18.4)	753 (18.1)
D/E	278 (25.4)	41 (24.9)	1040 (25.1)
**Region in England, *n* (%)**			
North	328 (29.9)	41 (25.1)	1150 (27.7)
Midlands	321 (29.3)	42 (25.4)	1259 (30.3)
South	282 (25.7)	48 (29.2)	1101 (26.5)
London	165 (15.1)	33 (20.3)	642 (15.5)
**Cohabitation, *n* (%)**			
Married/cohabitating	698 (63.7)	105 (64.1)	2348 (56.5)
Single	250 (22.8)	30 (18.5)	1096 (25.8)
Separated/widowed/divorced	148 (13.5)	29 (17.4)	736 (17.7)
**Level of education, *n* (%)**			
GCSE equivalent (school to 16 years)	248 (22.6)	37 (22.7)	1033 (24.9)
A-level equivalent (school to 18 years)	251 (22.9)	40 (24.3)	917 (22.1)
Degree equivalent (college/university)	372 (34.0)	53 (32.0)	1276 (30.7)
No formal qualifications	225 (20.6)	35 (21.0)	927 (22.3)
**Employment type, *n* (%)**			
Full-time employment	355 (32.4)	50 (30.6)	1111 (26.8)
Part-time employment	243 (22.1)	29 (17.6)	846 (20.4)
Self-employed	61 (5.6)	7 (4.1)	228 (5.5)
Other/not employed	437 (39.9)	78 (47.8)	1968 (47.4)
**Children aged <17 years in the household, *n* (%)**			
None	708 (64.6)	107 (65.4)	3044 (73.3)
1	225 (20.6)	27 (16.6)	638 (15.4)
2	115 (10.5)	20 (11.9)	368 (8.9)
≥3	47 (4.3)	10 (6.1)	104 (2.5)

GCSE = General Certificate of Secondary Education. UTI = urinary tract infection.

The *n* value was rounded to the nearest whole number, which accounts for slight discrepancies in associated proportions.

Of women who had UTI symptoms in the previous year, 47% (*n* = 510) of women sought care from a doctor or nurse at a local practice; 5% (*n* = 55) from an out-of-hours GP or nurse; 7% (*n* = 76) from accident and emergency, urgent care, or walk-in centre; 16% (*n* = 176) contacted a community pharmacist; and 22% (*n* = 235) of women did not seek care. Most women who consulted a healthcare professional (not including community pharmacy) used a phone 67% (*n* = 461); 16% via in-person consultation (*n* =112); 8% (*n* = 58) via e-form; 6% (*n* = 41) via web chat; 6% (*n* = 41) via non-video internet call; and 5% (*n* = 34) via video call (Supplementary Table 1). Only 16% (*n* = 112) reported an in-person consultation for their UTI symptoms (Supplementary Table 1).

### Women’s backgrounds

Women had increased odds of reporting UTI symptoms in the previous year if they had completed college or university compared with early secondary education (AOR = 1.30, 95% CI = 1.07 to 1.58); had ≥1 children under 17 years in the household (AOR = 1.58, 95% CI = 1.29 to 1.94); or ≥3 children under 17 years in the household (AOR = 2.45, 95% CI = 1.61 to 3.74), compared with having no children (Table 2).

**Table 2.** Women’s background characteristics (*n* = 4153) and AOR of UTI reporting in the previous year (weighted)

	≥1 UTI in the previous year(*n* = 1096)			≥3 UTIs in the previous year (recurrent)(*n* = 164)		
	AOR	(95% CI)		AOR	(95% CI)	
**Age, years**						
45–65	0.77	(0.64 to 0.93)		0.89	(0.57 to 1.40)	
>65	0.64	(0.49 to 0.83)		0.97	(0.52 to 1.81)	
<45	1			1		
**Social grade**						
C1	1.08	(0.89 to 1.31)		0.89	(0.56 to 1.41)	
C2	0.95	(0.76 to 1.18)		1.05	(0.62 to 1.78)	
D/E	1.13	(0.92 to 1.40)		0.94	(0.57 to 1.56)	
A/B	1			1		
**Region in England**						
Midlands	0.88	(0.73 to 1.06)		0.98	(0.61 to 1.58)	
South	0.87	(0.72 to 1.06)		1.45	(0.91 to 2.29)	
London	1.02	(0.80 to 1.29)		1.68	(0.97 to 2.90)	
North	1			1		
**Marital status**						
Single	0.66	(0.55 to 0.80)		0.81	(0.50 to 1.30)	
Separated/widowed/divorced	0.70	(0.57 to 0.88)		1.32	(0.78 to 2.21)	
Married/cohabitating	1			1.0		
**Education**						
A-level equivalent (school to 18 years)	1.13	(0.91 to 1.40)		1.13	(0.68 to 1.85)	
Degree equivalent (college/university)	1.30	(1.07 to 1.58)		0.89	(0.55 to 1.43)	
No formal qualifications	1.24	(0.99 to 1.54)		0.98	(0.58 to 1.67)	
GCSE equivalent (school to 16 years)	1			1		
**Employment**						
Part-time employment	0.86	(0.70 to 1.05)		0.76	(0.46 to 1.28)	
Self-employed	0.81	(0.58 to 1.14)		0.70	(0.29 to 1.71)	
Other/not employed	0.70	(0.57 to 0.84)		1.23	(0.79 to 1.92)	
Full-time employment	1			1		
**Children aged under 17 in household, *n***						
1	1.58	(1.29 to 1.94)		0.80	(0.48 to 1.32)	
2	1.19	(0.91 to 1.54)		1.24	(0.68 to 2.25)	
≥3	2.45	(1.61 to 3.74)		1.54	(0.70 to 3.40)	
None	1			1		
**Ethnic group**						
White, non-UK	0.63	(0.47 to 0.85)		0.94	(0.46 to 1.90)	
Mixed ethnic group	1.13	(0.66 to 1.91)		0.60	(0.14 to 2.60)	
Asian	0.66	(0.46 to 0.95)		1.65	(0.76 to 3.59)	
Black	0.50	(0.29 to 0.85)		1.72	(0.57 to 5.19)	
Other	1.28	(0.55 to 2.99)		2.34	(0.56 to 9.76)	
No answer	0.18	(0.08 to 0.40)		0.92	(0.10 to 8.62)	
White, UK	1			1		

AOR = adjusted odds ratio. CI = confidence interval. GCSE = General Certificate of Secondary Education. UTI = urinary tract infection.

Twenty-seven observations where women were unsure when their UTI was in the previous 12 months were excluded. Adjusted for age, ethnicity, social grade, region, marital status, educational status, employment, and number of children in household.

Women had reduced odds of reporting UTI symptoms in the previous year if they were aged 45–64 years (AOR = 0.77, 95% CI = 0.64 to 0.93) or >65 years (AOR = 0.64, 95% CI = 0.49 to 0.83) compared with those aged <45 years. Women who were 'Other/not employed(AOR = 0.70, 95% CI = 0.57 to 0.84) also had reduced odds of UTI reporting compared with those in full-time employment, as did those who were single (AOR = 0.66, 95% CI = 0.55 to 0.80) or separated, widowed, or divorced (AOR = 0.70, 95% CI = 0.56 to 0.88) compared with those married or cohabitating. Some ethnic minority groups also reported lower odds of UTI when compared with women who identified as White women from the UK (Table 2).

There were not significant associations noted between recurrent UTI reporting and background characteristics.

### Symptoms

Within the sample (*n* = 1096), frequency (65%) and dysuria (65%) were the most reported symptoms (Table 3); 19% of women reported having vaginal discharge; 47% of women reported ≥2 of the 'strongly predictive symptoms'; and 39% reported 'systemic upset' symptoms (Table 3).

**Table 3.** AOR for symptom reporting and odds of urine sampling, antibiotic prescribing, and immediate or delayed antibiotic prescriptions with 95% CIs, weighted (*n* = 1096)

Symptom reported	*n* (%)	Provided a urine sample for most recent UTI (*n* = 499)	Prescribed an antibiotic for most recent UTI (*n* = 691)	Given a back-up/stand-by antibiotic (*n* = 69) compared with immediate antibiotic (*n* = 616)
		AOR (95% CI)	AOR (95% CI)	AOR (95% CI)
**Nocturia**	515 (47)	1.10 (0.85 to 1.41)	1.13 (0.87 to 1.46)	0.44 (0.24 to 0.81)
**Cloudy urine**	407 (37)	1.25 (0.97 to 1.62)	1.15 (0.88 to 1.50)	0.54 (0.28 to 1.03)
**Dysuria**	713 (65)	0.50 (0.38 to 0.65)	0.65 (0.49 to 0.85)	0.74 (0.42 to 1.29)
**Frequency**	715 (65)	0.57 (0.44 to 0.75)	0.63 (0.48 to 0.83)	0.60 (0.34 to 1.07)
**Haematuria**	414 (13)	1.54 (1.05 to 2.24)	2.81 (1.79 to 4.41)	1.15 (0.55 to 2.41)
**Incontinence**	157 (14)	1.00 (0.70 to 1.44)	0.98 (0.68 to 1.41)	2.38 (1.08 to 5.25)
**Pain in lower abdomen**	549 (50)	1.09 (0.85 to 1.40)	1.35 (1.04 to 1.74)	0.53 (0.30 to 0.95)
**Vaginal discharge**	207 (19)	0.79 (0.56 to 1.10)	0.69 (0.50 to 0.96)	1.01 (0.49 to 2.06)
**Increased confusion**	63 (6)	1.27 (0.74 to 2.18)	2.14 (1.16 to 3.94)	2.90 (1.26 to 6.69)
**Unsteady on feet**	95 (9)	1.14 (0.73 to 1.78)	1.05 (0.67 to 1.65)	2.50 (1.13 to 5.54)
**Lower temperature**	46 (4)	1.06 (0.55 to 2.03)	1.40 (0.71 to 2.75)	3.08 (1.24 to 7.63)
**Fever**	146 (13)	2.49 (1.69 to 3.65)	2.10 (1.39 to 3.18)	1.44 (0.73 to 2.86)
**Rigours/shivering/ shaking**	109 (10)	1.50 (0.98 to 2.29)	1.70 (1.08 to 2.68)	1.46 (0.67 to 3.16)
**Kidney pain**	279 (25)	1.28 (0.96 to 1.70)	1.66 (1.22 to 2.25)	0.63 (0.31 to 1.26)
**Systemic symptoms**				
Any of low temperature, fever, rigours, or kidney pain	424 (39)	1.66 (1.28 to 2.15)	2.04 (1.56 to 2.69)	0.90 (0.50 to 1.60)
**Strongly predictive**				
>2 cloudy urine, nocturia, dysuria	510 (47)	0.80 (0.63 to 1.03)	0.95 (0.74 to 1.23)	0.51 (0.27 to 0.94)
**Recurrent UTI**				
>3 UTIs in last year	164 (15)	1.12 (0.79 to 1.59)	1.34 (0.93 to 1.94)	2.97 (1.53 to 5.78)

AOR = adjusted odds ratio. CI = confidence interval. UTI = urinary tract infection.

Adjusted for age, ethnicity, social grade, region, marital status, educational status, employment, and number of children in household.

Forty-six percent (*n* = 499) of women provided a urine sample for testing; odds of providing a sample were higher if they had haematuria (AOR = 1.54, 95% CI = 1.05 to 2.24), fever (AOR = 2.49, 95% CI = 1.69 to 3.65), or systemic upset (AOR = 1.66, 95% CI = 1.28 to 2.15). Women who reported higher severity had higher odds of providing a urine sample (AOR = 1.59, 95% CI = 1.26 to 2.0) (Table 4). Women with dysuria (AOR = 0.50, 95% CI = 0.38 to 0.65) or frequency (AOR = 0.57, 95% CI = 0.44 to 0.75) had reduced odds of urine sample provision.

**Table 4.** The AORs of increasing symptom severity and management outcomes, weighted

		Severity score *n* (%)0 is lowest severity and 10 is highest severity											AOR(95% CI)
		0	1	2	3	4	5	6	7	8	9	10	
**Provided urine sample for most recent UTI**	Yes (*n* = 497)	2%	1%	3%	4%	7%	13%	18%	22%	18%	7%	6%	1.59 (1.26 to 2.0)
	No (*n* = 569)	–	1%	3%	9%	8%	16%	21%	23%	12%	5%	2%	Ref
**Prescribed antibiotic for most recent UTI**	Yes (*n* = 691)	1%	1%	2%	4%	6%	14%	18%	23%	19%	7%	6%	2.24 (1.76 to 2.86)
	No (*n* = 404)	1%	2%	4%	11%	10%	15%	23%	20%	9%	4%	1%	Ref
**Given a back-up/stand-by antibiotic vs immediate antibiotic**	Delayed (*n* = 69)	1%	–	5%	4%	11%	13%	19%	21%	14%	11%	2%	0.74 (0.45 to 1.21)
	ASAP (*n* = 615)	1%	1%	2%	4%	5%	13%	18%	24%	19%	7%	6%	Ref

AOR = adjusted odds ratio. CI = confidence interval. GCSE = General Certificate of Secondary Education. UTI = urinary tract infection.

Adjusted for age, ethnicity, social grade, region, marital status, educational status, employment, and number of children in household.

Sixty-three percent (*n* = 691) of women reported receiving an antibiotic prescription; the odds of receiving an antibiotic increased if the woman reported haematuria (AOR = 2.81, 95% CI = 1.79 to 4.41), lower abdominal pain (AOR = 1.35, 95% CI = 1.04 to 1.74), confusion (AOR = 2.14, 95% CI = 1.16 to 3.94), flank pain (AOR = 1.66, 95% CI = 1.22 to 2.25), fever (AOR = 2.10, 95% CI = 1.39 to 3.18), rigours/shivering (AOR = 1.70, 95% CI = 1.08 to 2.68), kidney pain (AOR = 1.66, 95% CI = 1.22 to 2.25), or any one sign or symptom of systemic upset (AOR = 2.04, 95% CI = 1.56 to 2.69). Women who reported having higher symptom severity had higher odds of receiving an antibiotic (AOR = 2.24, 95% CI = 1.76 to 2.86). Women with dysuria (AOR = 0.65, 95% CI = 0.49 to 0.85), frequency (AOR = 0.63, 95% CI = 0.48 to 0.83), or vaginal discharge (AOR = 0.69, 95% CI = 0.50 to 0.96) reported lower odds of receiving an antibiotic.

Of women who received an antibiotic, 89% (*n* = 616) reported an immediate prescription and 10% (*n* = 69) reported a back-up, delayed, or stand-by prescription ('delayed prescription'). Women had lower odds of reporting a delayed antibiotic (compared with immediate) if they had nocturia (AOR = 0.44, 95% CI = 0.24 to 0.81), abdominal pain (AOR = 0.53, 95% CI = 0.3 to 0.95), or strongly predictive symptoms (AOR = 0.51, 95% CI = 0.27 to 0.94) (Table 3). The odds of reporting a delayed antibiotic increased if women had incontinence, (AOR = 2.38, 95% CI = 1.08 to 5.25), confusion (AOR = 2.90, 95% CI = 1.26 to 6.69), unsteadiness (AOR = 2.50, 95% CI = 1.13 to 5.54), or low temperature (AOR = 3.08, 95% CI = 1.24 to 7.63). Women had increased odds of receiving a delayed prescription if they had recurrent UTI (AOR = 2.97, 95% CI = 1.53 to 5.78).

**Figure 1.**
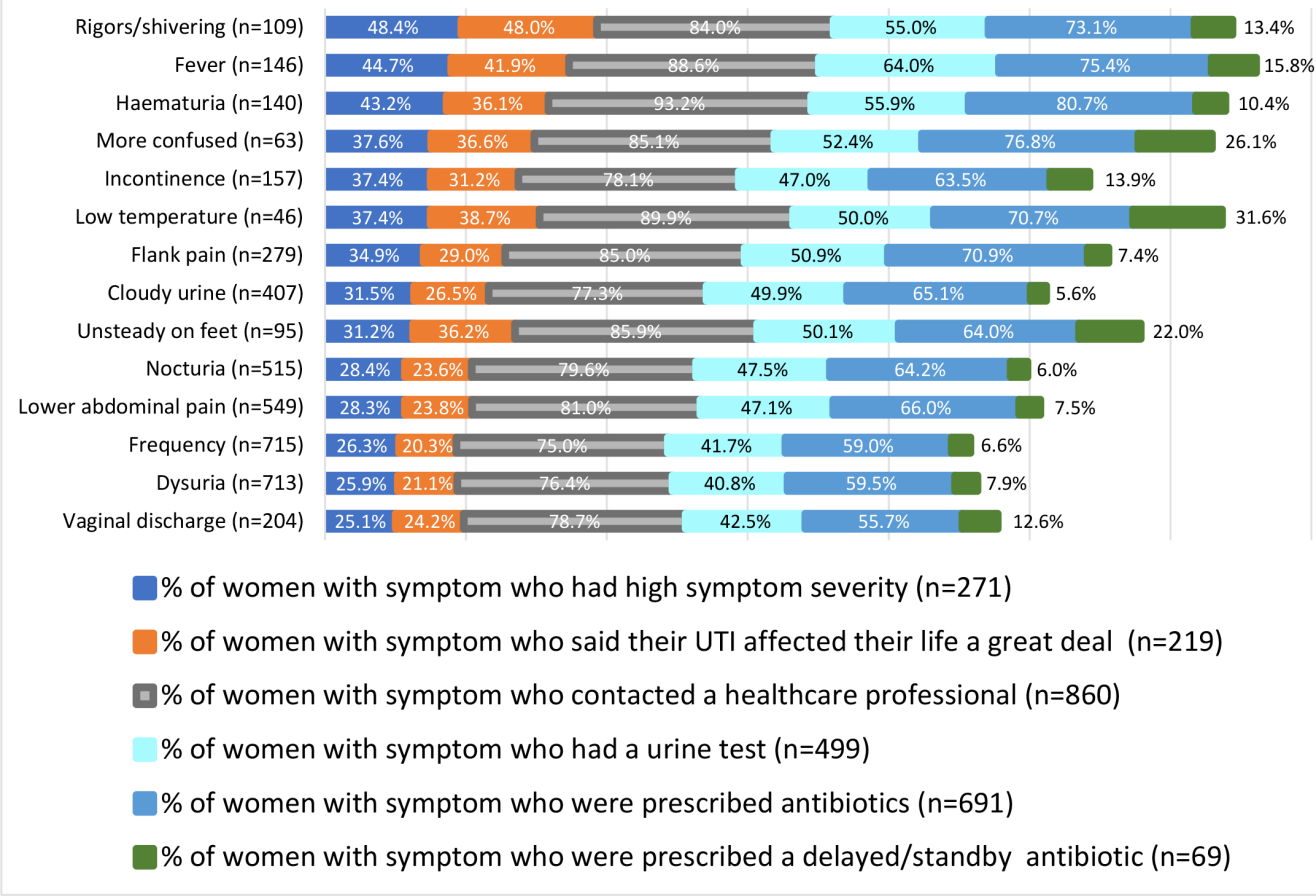
Symptom severity and management outcomes as a percentage of women with UTI signs and symptoms in the previous year. UTI = urinary tract infection. [Colours running left to right across the figure run top to bottom in the key; for example, rightmost colour on the figure is bottom in the key]

Proportionately, women with symptoms like vaginal discharge, dysuria, and frequency felt lower symptom severity or impact on quality of life; sought care less often; and reported having a urine test or receiving an antibiotic less frequently (Figure 1).

## Discussion

### Summary

This study highlights aspects of women’s backgrounds that may influence reporting of UTI symptoms, including age group, marital status, and having children in the household. By including a wide range of symptoms and women who choose not to seek health care, this study included a cohort of women who are often missed in studies focusing on UTI diagnosis and management. Findings showed that, though most consultations were conducted via phone, clinical outcomes aligned generally with national guidance for UTI management in England. In many cases, women’s perception of symptoms showed a strong correlation with management outcomes, but findings also showed areas where current guidance could be developed further (Table 5).

**Table 5.** Key findings and recommendations in relation to UTI prevention, diagnosis, and management guidance in England

Guideline recommendation for women aged <65 years with lower UTI^3,21,22^	Study findings and implications	Recommendation for future guidance and resource development
Consider alternative diagnosis if women have vaginal discharge	Odds of receiving antibiotics was significantly protective if responder reported vaginal discharge, which could show adherence to national guidance and/or reflect the finding that women with vaginal discharge were proportionately less likely to rate their symptoms as severe or seek care when compared with other symptoms.	Continue to indicate that women with vaginal discharge should be assessed for alternative diagnosis.
If signs and symptoms of pyelonephritis, send urine for culture and immediately start antibiotics.	Odds of urine samples being sent and prescribing of antibiotics were significantly higher with fever and other symptoms of systemic upset, aligning with national prescribing guidance.Odds of delayed antibiotic use for systemic symptoms were not significant.	Ensure that symptoms of pyelonephritis and sepsis are clearly outlined and linked to management guidance.
2 or 3 of dysuria, nocturia, cloudy urine (predictive symptoms): urine dipstick not needed to confirm diagnosis.1 of dysuria, nocturia, cloudy urine or any of urgency, frequency, haematuria, suprapubic tenderness (urinary symptoms): sample needed for dipstick.	Unclear in analysis if the sample was meant for urine dipstick or other analysis.2 of 3 key diagnostic symptoms was not protective of sampling. This could be related to sample collection for reasons beside urine dipstick (eg, culture/sensitivity).More likely to have urine sampled if haematuria (apart for systemic symptoms discussed above) which follows guidance and could also link to concerns of other diagnosis.Women with dysuria and frequency were less likely to provide a urine sample, which could link to perceived perception about the predictability of these symptoms by the clinician, symptom severity, and care seeking (women with these symptoms were less likely to seek care).	Provide guidance on symptoms that are diagnostically predictive as remote consultation can impact sampling as a tool for diagnosis.
≥2 predictive symptoms or other urinary symptoms and a positive dipstick for nitrites OR leukocytes with RBCs: if mild symptoms, watch and wait with back-up antibiotic OR consider immediate antibiotic.Urinary symptoms and positive dipstick for leukocytes: consider immediate or back-up antibiotic depending on symptom severity.Other or no symptoms and/or negative dipstick results: reassure that UTI is unlikely, consider other diagnosis and safety net.	Predictive symptoms were not correlated with any prescribing, but were linked to higher odds of delayed prescribing.Dysuria and frequency were protective against prescribing, which may be partially explained by proportionately lower symptom severity and care seeking in these groups.Confusion, haematuria, and lower abdominal pain were linked to higher odds of prescribing, which could in part also be explained by proportionately higher severity and care seeking.Strongly predictive symptoms, nocturia, and abdominal pain were protective against delayed prescribing, but women with more non-specific symptoms like confusion and incontinence had a higher odds of delayed prescription, following an expected trend.	Continue to provide guidance on decisionmaking for key diagnostic symptoms.Continue to ensure that symptom severity is captured in diagnostic guidance decision-making tools.Ensure diagnostic guidance provides avenues for management of symptoms of UTI that are not commonly reported in diagnostic studies. This includes less specific symptoms like incontinence, which was reported across age cohorts in this study.

RBC = red blood cell. UTI = urinary tract infection.

### Comparison with existing literature

In this study, women reported more phone consultations than in-person appointments for UTI (63% vs 16%, respectively). This aligns with other research using data from NHS England showing that, from 1 April to 31 Aug 2020, face-to-face consultations in primary care decreased by about 52% and phone appointments increased by 270% (compared with pre-pandemic levels), possibly contributing to an increased rate of antimicrobial prescribing at the start of the pandemic.^9^ However, subsequent data show that although rates of prescribing have decreased to pre-pandemic levels, phone consultations in England remain higher than pre-pandemic levels (31.6% in July 2022, compared with 14.2% in July 2019).^10^ Although this indicates that phone consultations are not causing a significant increase in antimicrobial prescribing for infections in a post-pandemic context, it highlights the continued need for clinicians to be supported in their management of infections like UTIs through multiple types of consultation.

Understanding how women’s backgrounds and lifestyles influence their risk of UTI, and how they choose to seek care for UTI, can help ensure the right messages and interventions to both prevent and manage infections are successfully developed and targeted. This study's findings showed increased odds of UTI reporting in women 16–45 years compared with women in older age groups, and in married or cohabitating women compared with those who were single. Sexual intercourse may trigger UTI in some women.^11^ Early research on sexual habits during the first 4 months of the COVID-19 lockdown in Britain showed that the rate of partnered sex in lockdown was highest in women aged 25–44 years. Women living without a partner were more likely to report a decrease in sexual intercourse, while those cohabitating reported no significant change in habits.^12^ Sexual habits may partly explain the relationship between age group, marital status, and UTI reporting seen in this study, and further highlight the importance of sexual intercourse as a trigger for UTI. Using patient-facing leaflets and resources to discuss UTI prevention with women may help reinforce these messages during and after consultations.^13,14^

Data also showed a relationship between number of children in the household and UTI reporting, even when age and marital status were accounted for in the model. There is little published literature linking parity and UTI risk. Kline et al conducted a study in 2014 assessing the characteristics of mice with two types of pathogenic UTI.^15^ In aged mice, multiparity increased susceptibility to acute cystitis or pyelonephritis and chronic infection when compared with nulliparous mice. It is known that childbirth can be associated with changes to the urinary tract (like incontinence) that may affect UTI susceptibility, further highlighting the importance of sharing key messages specific to UTI prevention at this time.^16^

Odds were protective against prescribing for women with vaginal discharge, aligning with national management guidance in the UK.^3^ Although dysuria and frequency are considered diagnostically predictive symptoms for UTI in a clinical setting, women with these symptoms reported less urine testing and antibiotic prescriptions. The latter is surprising given their prominence in national diagnostic guidance.^7,8^ One explanation may be that proportionately fewer woman with any one of these symptoms reported high symptom severity or contacted a healthcare provider (Figure 1). Alternatively, women with symptoms like visible haematuria and systemic upset reported proportionately higher symptom severity, higher levels of care seeking, and increased odds for urine sampling and prescribing. These trends could show that women feel more comfortable self-managing mild symptoms traditionally equated with a UTI (like dysuria and frequency). However, women may become more concerned with symptoms that influence their quality of life, or that they link with more serious conditions like sepsis or cancer.

It makes sense that symptom severity influenced whether women sought care and how that care was managed. A 2019 case control study compared Acute Cystitis Symptom Scores tools across multiple countries assessing symptom severity and the odds of physician-confirmed UTI diagnosis (based on history, urine tests, and the national guidance).^17^ Findings showed that an increase in symptom severity improved the diagnostic ORs significantly for multiple symptoms including frequency, urgency, dysuria, abdominal pain, haematuria, and flank pain. Findings from an English study aiming to develop clinical guidelines to predict UTI (based on urine culture) showed that symptoms with lower severity were less predictive; however, when testing this, symptom groupings were found to be more accurate at detecting a positive culture when key symptoms of any severity were included.^7^ A meta-analysis that analysed UTI management studies supporting delayed or non-prescribing interventions assessed the impact of symptom severity on outcomes, and found that individuals in the highest symptom severity category had slightly lower odds of full clinical recovery if they received the intervention (OR = 0.991, 95% CI = 0.983 to 0.999), but they were unable to confirm a link to UTI diagnosis.^18^ Though symptom severity is part of the diagnostic process, both from a care-seeking and clinical standpoint, further research is needed clarify how it should be used as part of decisionmaking in UTI guidance; specifically regarding how symptom severity equates to management and then clinical outcomes.

### Strengths and limitations

Strengths of this study include a large sample size that was weighted to allow for broad representation of the household population. Using an e-survey may have allowed women to report about UTIs more openly than data collected by an interviewer. UTI reporting was higher in this study than in a previous face-to-face study (11% in 2014 compared with 26% in 2021), but it is unclear if this is related to the context or methodology.^4^ An e-survey conducted in The Netherlands in early 2020 found similarly high levels of reporting, where 533 out of 975 responders with 'any UTI' had a UTI in the previous year (55%).^19^ Because UTI is self-reported and not based on clinical diagnosis in the present study, it may mean that some women had an alternative diagnosis and UTI was over-reported. However, evidence has shown that women with frequency or dysuria are generally able to self-diagnose a UTI.^20^ Women who have experience with UTIs may have self-selected for this e-survey, or women may have been more willing to reflect upon and report on a sensitive topic like UTIs without a facilitator present, leading to higher reporting prevalence. This study excluded women less able to access the internet, biasing selection for some key groups; for instance, women living with some disabilities or those living in care homes. Because this study was conducted over the first year of the COVID-19 pandemic, lockdown context and prescribing thresholds influenced prescribing and other management behaviours.

### Implications for research and practice

These findings link a woman’s background characteristics to her development of UTI symptoms in the previous year, and highlight how the commencement of sexual activity and childbirth may be key times to target women with messages specific to UTI prevention. Findings in relation to UTI clinical guidance highlight the importance of including information on key symptoms (including non-specific symptoms) to guide diagnosis and management, especially considering an increase in remote consultations. Though women’s symptoms guide management outcomes, perceived severity of women’s infection is significantly linked to these outcomes, highlighting the need to clarify how to use severity as part of the decision-making process in current guidance and conduct further research into understanding how symptom severity links to UTI diagnosis, urine culture, and clinical recovery.

## Data

The dataset relied on in this article is available from the corresponding author on reasonable request.
